# Modeling and Prediction of Wearable Energy Harvesting Sliding Shoes for Metabolic Cost and Energy Rate Outside of the Lab

**DOI:** 10.3390/s20236915

**Published:** 2020-12-03

**Authors:** Peter B. Shull, Haisheng Xia

**Affiliations:** 1 The State Key Laboratory of Mechanical System and Vibration, School of Mechanical Engineering, Shanghai Jiao Tong University, Shanghai 200240, China; pshull@sjtu.edu.cn; 2 Department of Automation, University of Science and Technology of China, Hefei 230026, China

**Keywords:** wearable device, energy harvesting, metabolic cost, gait, model prediction

## Abstract

The recent explosion of wearable electronics has led to widespread interest in harvesting human movement energy, particularly during walking, for clinical and health applications. However, the amount of energy available to harvest and the required metabolic rate for wearable energy harvesting varies across subjects. In this paper, we utilize custom energy harvesting sliding shoes to develop and evaluate multivariate linear regression models to predict metabolic rate and energy harvesting rate during overground walking outside of the lab. Subjects performed 200 m self-selected normal and fast walking trials on flat ground with custom sliding shoes. Metabolic rate was measured with a portable breathing analysis system and energy harvesting rate was measured directly from the generator on the custom sliding shoes. Model performance was determined by comparing the difference between actual and predicted metabolic and energy harvesting rates. Overall, predictive modeling closely matched the actual values, and there was no statistical difference between actual and predicted average metabolic rate or between actual and predicted average energy harvesting rate. Energy harvesting sliding shoes could potentially be used for a variety of wearable devices to reduce onboard energy storage, and these findings could serve to inform expected energy harvesting rates and associated required metabolic cost for a diverse array of medical and health applications.

## 1. Introduction

The increase in wearable electronics for clinical and health applications [1,2] has led to widespread research interest in harvesting human movement energy to potentially power these electronics. There are two primary approaches to harvesting human energy: inertial energy harvesting and direct force energy harvesting. For inertial energy harvesting, human motion induces cyclical movements in the proof mass of a device worn on the body. Mechanical energy from the movements is then converted to electrical energy such as for a magnet moving back and forth through a coil [3] or deflections in a piezoelectric suspension beam [4]. A benefit of this approach is that because energy is harvested indirectly from vibratory movements on the skin or clothes, it does not typically increase metabolic cost during human movement. However, a significant drawback is that inertial energy harvesting only captures relatively small amounts of power, typically on the order of a few mW or less [5,6,7]. For low power situations this may be acceptable, though for most wearable applications involving sensors or actuators it is insufficient [8], and in this context wireless communication typically constitutes the largest part of energy expenditure.

Direct force energy harvesting, in contrast, occurs as mechanical human movement forces are directly converted to electrical power, which can result in higher energy harvesting rates [9]. The most common approach is to capture energy from the vertical force of the heel as it contacts the ground during walking gait, such as through a metal spring with a coupled generator [10], a stack of electroactive polymers [11], or a trapezoidal slider mechanism [12]. Other approaches include a powered knee-brace device that harvests energy during knee extension [13] and a suspended-load backpack that converts mechanical energy from vertical movements of the carried load into electricity [14].

Metabolic rate tends to increase for direct force energy harvesting due to the elevated physical effort required [15]. For example, harvesting energy with a powered knee-brace device caused metabolic cost to increase by 18 W during continuous motion generation mode and by 5 W during generative braking mode [13]. Similarly, harvesting energy with a suspended-load backpack caused metabolic rate to increase by 19 W [14]. Depending on the degree of increase and the specific application of operation, increased metabolic rate could be a deterrent to the adoption of wearable energy harvesting devices, thus it is important to characterize metabolic rate while walking with energy harvesting devices.

In addition to the presence of energy harvesting, several other primary factors affect metabolic rate during gait, including height, weight, walking speed, and step frequency. Height, which is directly proportional to leg length, influences metabolic rate and is thus a determining factor in optimal walking speed selection in humans and other mammals [16]. Weight is also directly related to metabolic rate; increased body weight requires a higher metabolic rate and reduced weight requires a lower rate [17,18]. Similarly, walking speed has been shown to directly influence metabolic rate; increased walking speed leads to a higher metabolic rate and decreased walking speed leads to a lower rate [19]. Step frequency also plays a significant, independent role in changing metabolic rates [20]. In one study, smart shoes based on embedded strain sensors were developed to classify gait as normal, in-toeing, or out-toeing and to directly estimate energy consumption [21]. For energy harvesting, the major influencing factors include when and how long the harvester is activated during each gait cycle, as well as walking speed and step frequency [13]. Since metabolic and energy harvesting rates are vital to practical applications of wearable energy harvesting, it is critical to understand and predict these quantities to inform design and practical implementation.

Predictive modelling has been applied to a variety of biomechanical applications via various machine learning techniques including to predict early signs of Parkinson’s disease [22], to detect falls and freezing of gait [23,24] and for classifying general human physical activities [25]. However, it has not yet been utilized for direct force energy harvesting and metabolic rate estimation during gait for energy harvesting applications. Thus, the purpose of this work is to develop predictive modeling for metabolic rate and energy harvesting rate while walking with custom energy harvesting sliding shoes. These models could be specifically used to determine whether energy harvesting can produce enough energy to power one or more wearable electronic devices based on an individual’s height, weight, walking speed, and step frequency and to determine the associated metabolic rate and could generally serve to inform the scope and implementation of potential applications for energy harvesting to power wearable electronics.

## 2. Materials and Methods

### 2.1. Custom Sliding Shoe Design and Predictive Modeling 

**Figure 1:**
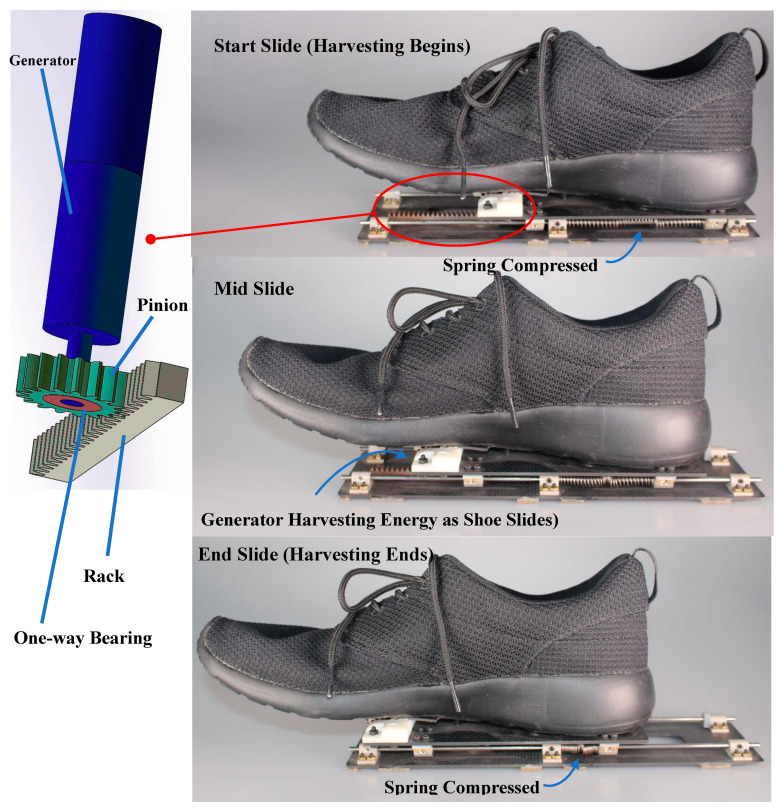
Custom sliding shoe created by mounting a sliding mechanism to the sole of a standard walking shoe. A rack-and-pinion converts linear sliding motion to rotational motion of the generator. Energy is harvested as the shoe slides during the stance phase of gait by the generator on the sliding mechanism. The compressed spring returns the sliding mechanism to the original position during the swing phase when the shoe is not in contact with the ground.

A custom sliding shoe was designed to harvest energy during walking gait [26]. A sliding mechanism was affixed to the sole of a standard walking shoe, and a generator (DC-5W, Maxon Motor, Sachseln, Switzerland) within the mechanism harvests energy during the stance phase of gait. A rack-and-pinion was used to convert linear sliding motion to rotational motion of the generator (Figure 1).

Energy harvesting begins at heel strike as the shoe slides and generates energy via the generator until mid-stance. The total sliding distance is 6.5 cm. The input shaft engages the generator during the stance phase as the shoe slides forward and disengages during the swing phase via a one-way bearing as the mechanism is pulled back to the original position via the compressed spring (Figure 1).

There are two plates in the sliding mechanism which are made of carbon fiber, and there are two steel rails to guide the linear sliding motion. The surface of the bottom plate that is in contact with the ground is covered in rubber tread to maintain stable ground contact. The top plate is attached to the sole of the shoe and connected to the bottom plate via the guide rails. The height and weight of the sliding mechanism are 2 cm and 288 g, respectively, and the overall weight of the sliding shoe system is 462 g. 

A multivariate linear regression model was developed to predict metabolic rate for subjects walking with the energy harvesting sliding shoes. The input features were subject height, weight, walking speed, and step frequency, and the output was predicted metabolic rate. The model is of the form: $\dot{m}=\theta_{0,m}+\theta_{1,m}x_{1}+\theta_{2,m}x_{2}+\theta_{3,m}x_{3}+\theta_{4,m}x_{4}$, where $\dot{m}$ is the metabolic rate, $x_{1}$, $x_{2}$, $x_{3}$, and $x_{4}$ are the features height, weight, walking speed, and step frequency, respectively, and $\theta_{0,m}$, $\theta_{1,m}$, $\theta_{2,m}$, $\theta_{3,m}$, and $\theta_{4,m}$ are the corresponding model weights.

A similar multivariate linear regression model was developed to predict the energy harvesting rate for subjects while walking with the custom sliding shoes. The input features were again subject height, weight, walking speed, and step frequency, and the output was the predicted energy harvesting rate. The model is of the form $\dot{e}=\theta_{0,e}+\theta_{1,e}x_{1}+\theta_{2,e}x_{2}+\theta_{3,e}x_{3}+\theta_{4,e}x_{4}$, where $\dot{e}$ is the energy harvesting rate, $x_{1}$, $x_{2}$, $x_{3}$, and $x_{4}$ are the features height, weight, walking speed, and step frequency, respectively, and $\theta_{0,e}$, $\theta_{1,e}$, $\theta_{2,e}$, $\theta_{3,e}$, and $\theta_{4,e}$ are the corresponding model weights. An extended energy harvesting model was also created based on the original model but also included the average maximum generator voltage per step as an additional feature. The extended model was developed for applications when the maximum voltage is easily determined during normal use. The developed metabolic rate and energy harvesting rate predictive models presented here are novel and distinct from previously proposed models on a similar research topic [27]. Specifically, because step frequency has been previously reported to independently influence metabolic rate and energy harvesting rate during walking gait [13,20], step frequency is included as an independent and key model parameter, and for energy harvesting the average maximum generator voltage in included in the extended model as a key feature for applications when this measurement is available to potentially improve model performance.

To determine model weights θ in the metabolic rate and energy harvesting multivariate linear regression models described above, for n subjects of experimental data, for each iteration i, x, $\dot{m}$, and $\dot{e}$ values were used from n − 1 subjects’ data to train the model weights for that iteration. This was repeated n times, leaving out a different subject’s data each iteration. Final model weights were then computed as the average weights for all n iterations. All computations were performed in Matlab, version 9.1 (Mathworks, Natick, MA, USA). The final model weights $\theta_{0,m}$, $\theta_{1,m}$, $\theta_{2,m}$, $\theta_{3,m}$, and $\theta_{4,m}$ for the predicted metabolic rate model were 168.42, −0.55, 2.42, −37.61, 243.98, respectively, and the final model weights $\theta_{0,e}$, $\theta_{1,e}$, $\theta_{2,e}$, $\theta_{3,e}$, and $\theta_{4,e}$ for the predicted energy harvesting rate model were −170.41, 2.30, −1.42, 63.13, and −100.35, respectively.

### 2.2. Experimental Protocol and Testing

Twelve healthy subjects (age: 26.6 ± 3.9 years, height: 172.5 ± 5.5 cm, mass: 66.3 ± 7.6 kg, all male) (Table 1) performed overground walking trials outside on flat ground with the custom sliding shoes to determine metabolic and energy harvesting rates to quantify modeling performance. Separate trials were also performed while subjects wore their own normal walking shoes to quantify baseline walking speeds, step frequencies, and metabolic rates. All subjects participated in this study after giving informed consent in accordance with the Declaration of Helsinki. Subjects were selected with foot sizes to comfortably fit in the size 42 EUR custom sliding shoes. 

**Figure 2:**
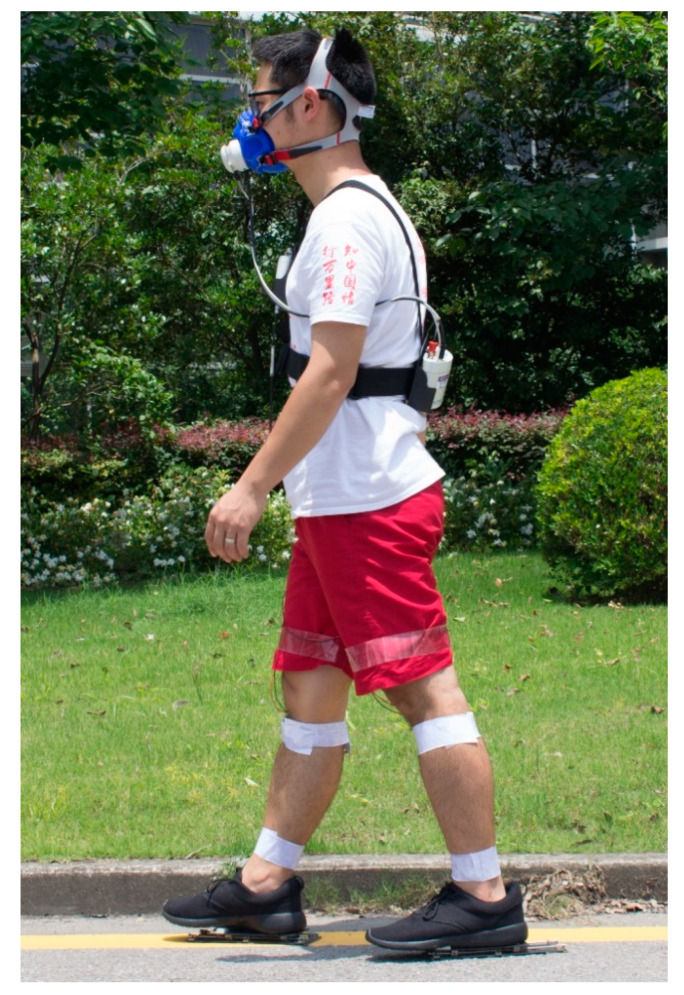
Experimental setup. Subjects performed 200 m normal and fast walking trials on flat ground with the custom sliding shoes. Metabolic rate was measured with a portable breathing analysis system and energy harvesting rate was measured from the generator on the custom sliding shoes.

To become accustomed to walking with the energy harvesting sliding shoes, subjects initially practiced walking on their own overground with the shoes for approximately 10 min prior to formal testing. After this, subjects performed a series of 4 different walking trials (Figure 2).

Each trial covered 200 m and was performed overground while walking in a straight line. Conditions of the walking trials were as follows:

(1) walking at a self-selected normal speed wearing the custom sliding shoes
(2) walking at a self-selected fast speed wearing the custom sliding shoes
(3) walking at a self-selected normal speed wearing their own normal walking shoes
(4) walking at a self-selected fast speed wearing their own normal walking shoes

Subjects performed walking trials in a randomized order and rested between each of the four trials for 3 min each or longer if requested. A portable intake system (K4b2, COSMED, Rome, Italy) was used to collect oxygen and carbon dioxide rates while breathing. These data were used to determine the metabolic rate while walking. Energy harvesting data were collected by sampling the voltage across a resistor in parallel with the energy harvesting generator at 100 Hz on the shoe via a data acquisition board (USB-1252A, Smacq, Shenzhen, China).

### 2.3. Data Analysis

Metabolic rate for each trial was calculated from the rate of oxygen consumption and carbon dioxide production with the respirometer using the Brockway equation [28]. Metabolic data from one minute preceding the last 30 s of each trial were used for analysis. Energy harvesting rate was computed for trials when subjects wore the custom sliding shoes as the average voltage across the resistor of the generator squared divided by resistance value. Walking speed was computed by dividing the 200 m walking distance by the time required to walk for each trial, and step frequency was computed as the number of steps taken divided by the time. Predicted metabolic rate and energy harvesting rate were computed as described above. Mean and standard deviation for metabolic rate and energy harvesting rate were computed across all subjects for each walking condition.

Model performance was determined by comparing the actual and predicted metabolic rates and energy harvesting rates via paired student’s t-test. Statistical significance was set to the level of *p* = 0.05. In addition, leave-one-out cross validation was performed n times for n subjects resulting in model errors for each iteration. Final aggregate percentage error and absolute error were computed as the average errors of all n iterations.

## 3. Results

**Figure 3:**
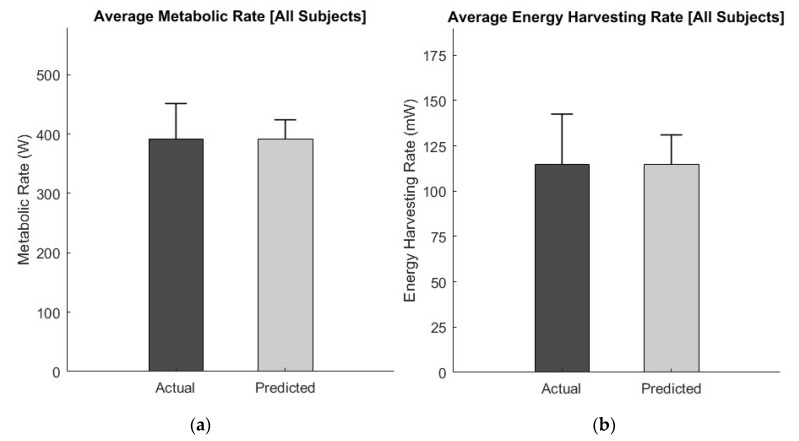
(**a**) Overall actual and predicted average metabolic rates. There was no statistical difference between actual and predicted average metabolic rates. (**b**) Overall actual and predicted average energy harvesting rates. There was no statistical difference between actual and predicted average energy harvesting rates.

Overall, predictive modeling closely matched actual values for both metabolic rate and energy harvesting rate (Figure 3).

**Figure 4:**
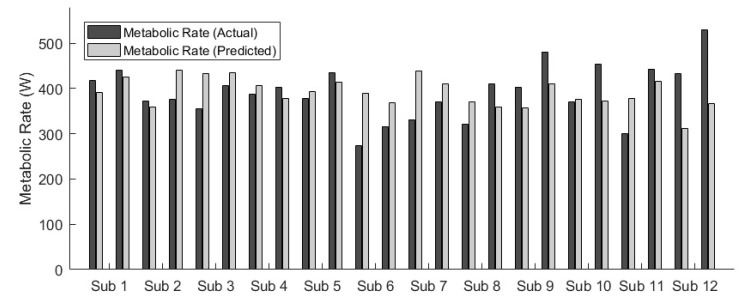
Metabolic rate modeling results across individuals. The trend of the predicted metabolic rates in general followed that of the actual metabolic rates across individual subjects.

There was no statistical difference between actual and predicted average metabolic rate (*p* = 0.99), and there was no statistical difference between actual and predicted average energy harvesting rate (*p* = 0.96). In addition, the trend of the predicted metabolic rates in general followed that of the actual metabolic rates across individual subjects (Figure 4).

**Figure 5:**
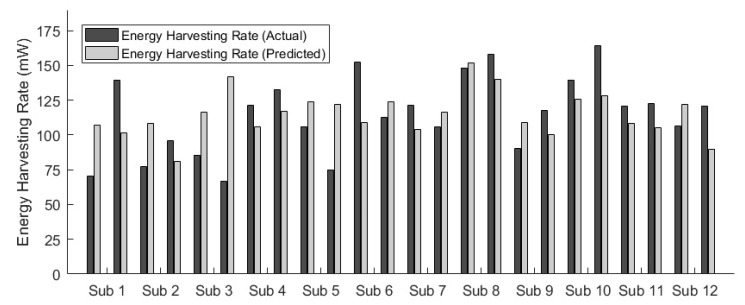
Energy harvesting rate modeling results across individuals. The trend of the predicted energy harvesting rates in general followed that of the actual energy harvesting rates across individual subjects.

Average absolute and percent errors for the metabolic rate model were 54.9 W and 14.4%, respectively. Similarly, the trend of the predicted energy harvesting rates in general followed that of the actual energy harvesting rates across individual subjects (Figure 5).

Average absolute and percent errors for the energy harvesting rate model were 24.6 mW and 24.9%, respectively. For the extended energy harvesting model, which included the additional average generator maximum voltage per step feature, the average absolute and percent errors were 15.1 mW and 13.8%, respectively.

For the normal walking speed trials with the custom sliding shoes, subjects chose a walking speed of 0.88 ± 0.15 m/s that resulted in a step frequency of 0.73 ± 0.10 steps/sec and an energy harvesting rate of 111.56 ± 26.06 mW. For the fast walking speed trials with the custom sliding shoes, subjects chose a walking speed of 1.10 ± 0.18 m/s that resulted in a step frequency of 0.86 ± 0.12 steps/sec and an energy harvesting rate of 117.63 ± 28.25 mW. The metabolic rates for the normal and fast walking speeds with the custom sliding shoes were 361.97 ± 45.69 and 422.33 ± 53.11 W, respectively. For the normal walking speed trials with their own walking shoes, subjects chose a walking speed of 1.24 ± 0.09 m/s that resulted in a step frequency of 0.92 ± 0.06 steps/sec. For the fast walking speed trials with their own walking shoes, subjects chose a walking speed of 1.54 ± 0.09 m/s that resulted in a step frequency of 1.01 ± 0.04 steps/sec. The metabolic rates for the normal and fast walking speeds with their own walking shoes were 305.87 ± 40.37 and 375.96 ± 43.10 W, respectively.

## 4. Discussion

This study presented predictive models for metabolic rate and energy harvesting rate for custom sliding shoes designed for harvesting energy during the stance phase of human gait. A strength of the models is that they only rely on the general subject features of height, weight, walking speed, and step frequency, which are relatively easy to ascertain in practical applications. Given the inherent complexity of both human metabolic rates [29] and energy harvesting [30], these results seem to provide a reasonable degree of predictive accuracy.

Adding additional features to these models could further improve the predictive ability, though this would come at the expense of potentially requiring increased effort to measure the additional features. In the presented model, it is relatively simple to measure the features of subject height, weight, walking speed, and step frequency. However, various commercial or custom wearable sensors [31,32] could enable a large array of additional gait features to potentially improve the models. For example, sensors can be embedded in the shoes or insoles to measure detailed foot characteristics during the stance and swing phases [33,34]. An ear-worn sensor could also be used to estimate temporal events and gaits asymmetries [35]. In the present study, adding an additional feature of the average maximum voltage of the generator on each step significantly improved the predictive ability of the energy harvesting rate model. Measuring this additional feature would, however, likely require significant hardware and software modifications for practical, widespread use. The tradeoffs of measuring additional features should be considered in future applications.

The results of using machine learning to develop predictive models for metabolic and energy harvesting rates in this study contribute to the growing body of work utilizing machine learning methods for wearable human movement applications. For example, machine learning techniques have been used with wearable sensors to estimate walking speed for individuals with multiple sclerosis [36], to predict early signs of Parkinson’s disease [22], and to detect falls and freezing of gait [23,24]. Most recent work, including the presented study, has relied on a relatively small set of training data to develop the predictive models. Since machine learning develops predictive models based on these training data, in general, the larger the dataset, the more robust the predictive models. Thus, future work involving wearables and machine learning should also consider testing more subjects to create larger datasets resulting in more robust models.

It appears that known trends for increasing walking speed with normal walking shoes may also be true for increasing walking speed with custom sliding shoes. In this study, while wearing the sliding shoes, fast walking resulted in increased step frequency and increased metabolic rate as compared with normal walking. These findings align with previous trends reported for normal walking shoes in which increased walking speed led to higher step rate [37] and higher metabolic rate [19] and also align with results for normal walking shoe testing in the presented study. Increased walking speed also increased the energy harvesting rate but only by a relatively small amount as compared to the required increase in metabolic rate. It may thus not be advantageous for users to increase walking speed as a means of increasing energy harvesting rate. Instead, extended training with the sliding shoes could enable users to increase energy harvesting rate as they learn to slide on each step during the stance phase more effectively and to simultaneously reduce metabolic rate as motor learning occurs requiring less effort.

While the trends for walking with custom sliding shoes and normal walking shoes at different speeds were similar, direct comparison between sliding and normal shoes were notably different. This discrepancy was likely due largely to the fact that subjects were not completely accustomed to walking with the custom sliding shoes. In this study, subjects were only given a single training block of about 10 min to practice walking with the custom sliding shoes. This was long enough to stably perform the 200 m straight line walking trials; however, it was likely not long enough to comfortably walk at normal speeds. Qualitatively, it appeared that subjects intently concentrated on walking movements, especially the new sliding motion during stance, and this concentration resulted in unconsciously walking more slowly than normal. Previous gait training research has shown that it may take six weeks of concentrated, weekly training sessions to adopt a new gait pattern [38,39]. Thus, it is possible that with extended gait training (e.g., for six weeks), subjects could become more familiar with the sliding shoes and increase their self-selected walking speeds to at or near the level of walking with normal shoes.

Metabolic rate was also significantly higher when walking with the custom sliding shoes as compared with normal walking shoes. While some of the increased rate can be attributed to the increase in energy required for harvesting energy by sliding the generator during stance, much of it is likely due to the learning of a new gait pattern which often involves elevated muscle co-contractions to increase stability while walking in a new and unfamiliar way. Over time, learning could enable users to feel more comfortable and to walking more stably with the custom sliding shoes, leading to reduced muscle co-contraction and lower metabolic rate.

A limitation of this study is that we did not perform long-term gait training with the custom sliding shoes. Long-term training could enable subjects to learn and adapt to the new motor pattern, which will potentially influence the metabolic rate and walking speed as detailed in the preceding paragraphs above. It is possible that the predictive models developed in this study would not performed as well for subjects walking with the custom sliding shoes after they have performed extended training, particularly for metabolic rate, because longer training times could result in increased energy harvesting rates and decreased metabolic rates. Another limitation is that only young, male subjects were tested. Given that age and sex play a significant role in human metabolism and the inherent musculoskeletal differences between genders and age, it is possible that the developed models would not perform as well for female subjects and for older subjects. Future research should focus on developing models to include these other important factors of age and sex to make the results more generalizable and widely applicable. In addition, we chose to use multivariate linear regression models in this study because they are relatively robust and produced reasonable results; however, exploring other machine learning regression algorithms such as polynomial regression, neural networks, or random forests could further improve the results. Thus, future work should focus on long-term gait training, including female subjects in the training set, and exploring other algorithms for developing the predictive models.

## 5. Conclusions

In this paper, we developed and quantified predictive models for estimating metabolic rate and energy harvesting rate while walking with energy harvesting sliding shoes. The models, based on the feature set of height, weight, walking speed, and step frequency, could potentially be used for future design and implementation of the energy harvesting sliding shoes in practical applications. For example, the energy harvesting model could be used in the design of wearable systems to determine what type and how many wearable sensors could be powered for a given subject’s profile and walking characteristics. It could also be used to determine how long someone would need to walk to charge a specific wearable device.

There are several potential applications of the energy harvesting custom sliding shoes. A significant portion of the world’s population lives without standard grid-based electricity and could potentially benefit from alternative forms of generating electricity for powering and charging lights and electronic devices. Applications involving walking or hiking in mountains or remote areas for extended periods of time could also benefit from a portable energy harvesting approach. It is possible that the custom sliding shoes could be used not only as a means of harvesting energy, but also to provide resistance training for exercise while walking. In this case, the metabolic rate model could be utilized to estimate the amount of required energy for a given walking speed and aid in helping subjects achieve specific exercise goals. For these and related applications, the presented predictive models could serve as a foundation for design and practical implementation.

## Figures and Tables

**Table 1:** Modeling parameters based on individual subject characteristics while walking with the custom sliding energy harvesting shoes.

Subject	Age (years)	Height (cm)	Weight (kg)	Walking Condition	Walking Speed (m/s)	Step Freq (Hz)	Metabolic Rate (W)	Energy Harvesting Rate (mW)
1	26	171	70	normal	0.87	0.75	418.0	70.2
				fast	1.24	0.94	439.8	139.5
2	28	160	53	normal	0.81	0.76	372.6	77.1
				fast	1.04	1.02	375.5	95.8
3	27	173	60	normal	1.01	0.96	354.8	85.6
				fast	1.36	1.06	406.4	66.7
4	24	169	69	normal	1.12	0.83	387.6	121.4
				fast	1.31	0.79	403.5	132.7
5	33	175	74	normal	1.08	0.74	377.5	106.1
				fast	1.19	0.86	435.8	75.1
6	24	171	60	normal	0.85	0.78	273.9	152.3
				fast	0.89	0.71	316.3	112.7
7	24	180	85	normal	0.81	0.64	331.5	121.2
				fast	0.85	0.63	371.1	105.8
8	36	183	65	normal	0.94	0.67	321.2	148.3
				fast	1.02	0.77	410.6	158.0
9	25	170	66	normal	0.67	0.63	402.9	90.2
				fast	1.05	0.91	480.8	117.6
10	25	175	65	normal	0.98	0.75	370.0	139.6
				fast	1.28	0.85	454.2	164.1
11	25	170	64	normal	0.78	0.69	300.8	120.5
				fast	1.14	0.94	443.4	122.6
12	22	173	65	normal	0.59	0.54	432.9	106.2
				fast	0.79	0.86	530.4	121.0
